# The Intensity-Modulated Pelvic Node and Bladder Radiotherapy (IMPART) Trial: A Phase II Single-Centre Prospective Study

**DOI:** 10.1016/j.clon.2019.07.017

**Published:** 2020-02

**Authors:** M.P. Tan, V. Harris, K. Warren-Oseni, F. McDonald, H. McNair, H. Taylor, V. Hansen, M. Sharabiani, K. Thomas, K. Jones, D. Dearnaley, S. Hafeez, R.A. Huddart

**Affiliations:** ∗ Academic Radiotherapy Unit, Institute of Cancer Research, Sutton, Surrey, UK; † The Royal Marsden NHS Foundation Trust, Sutton, Surrey, UK; ‡ Guy's & St. Thomas' NHS Foundation Trust, London, UK; § Laboratory of Radiation Physics, Odense University Hospital, Odense, Denmark; ¶ The School of Public Health, Imperial College London, London, UK; || ICBARC, London, UK

**Keywords:** Bladder cancer, IMRT, pelvic nodes, radiotherapy

## Abstract

**Aims:**

Node-positive bladder cancer (NPBC) carries a poor prognosis and has traditionally been treated palliatively. However, surgical series suggest that a subset of NPBC patients can achieve long-term control after cystectomy and lymph node dissection. There is little published data regarding the use of radiotherapy to treat NPBC patients. This is in part due to concerns regarding the toxicity of whole-pelvis radiotherapy using conventional techniques. We hypothesised that, using intensity-modulated radiotherapy (IMRT), the pelvic nodes and bladder could be treated within a radical treatment volume with acceptable toxicity profiles.

**Materials and methods:**

The Intensity-modulated Pelvic Node and Bladder Radiotherapy (IMPART) trial was a phase II single-centre prospective study designed to assess the feasibility of delivering IMRT to treat the bladder and pelvic nodes in patients with node-positive or high-risk node-negative bladder cancer (NNBC). The primary end point was meeting predetermined dose constraints. Secondary end points included acute and late toxicity, pelvic relapse-free survival and overall survival.

**Results:**

In total, 38 patients were recruited and treated between June 2009 and November 2012; 22/38 (58%) had NPBC; 31/38 (81.6%) received neoadjuvant chemotherapy; 18/38 (47%) received concurrent chemotherapy; 37/38 (97%) patients had radiotherapy planned as per protocol. Grade 3 gastrointestinal and genitourinary acute toxicity rates were 5.4 and 20.6%, respectively. At 1 year, the grade 3 late toxicity rate was 5%; 1-, 2- and 5-year pelvic relapse-free survival rates were 55, 37 and 26%, respectively. The median overall survival was 1.9 years (95% confidence interval 1.1–3.8) with 1-, 2- and 5-year overall survival rates of 68, 50 and 34%, respectively.

**Conclusion:**

Delivering IMRT to the bladder and pelvic nodes in NPBC and high-risk NNBC is feasible, with low toxicity and low pelvic nodal recurrence rates. Long-term control seems to be achievable in a subset of patients. However, relapse patterns suggest that strategies targeting both local recurrence and the development of distant metastases are required to improve patient outcomes.

## Highlights

• Radical IMRT to the bladder and pelvic nodes is feasible with low toxicity rates.
• A subset of node-positive bladder cancer patients attain disease control with IMRT.
• Relapse patterns suggest optimisation of local and systemic treatments is needed.

## Introduction

Regional lymph node involvement in bladder cancer is associated with a poor prognosis. Five-year disease-specific survival rates of 31.2% are reported compared with 66.7% in patients without nodal involvement [1]. The prognosis is so poor that in most centres, patients with node-positive bladder cancer (NPBC) have conventionally been considered to have metastatic disease, and as such have been treated with palliative intent. However, surgical series have shown that long-term control is achievable in a subset of patients after cystectomy and lymph node dissection [2], [3], [4], [5], [6] and, when combined with neoadjuvant chemotherapy, 5-year cancer-specific survival rates of up to 63.5% have been reported in those showing a complete pathological response after chemotherapy [7].

Although surgery has generally been the most common management of muscle-invasive bladder cancer (MIBC), bladder preservation strategies with chemoradiation have become an increasingly accepted alternative. The standard UK practice is to treat the whole bladder alone to a dose of 64 Gy in 32 fractions or equivalent using three-dimensional conformal radiotherapy. The pelvis is not routinely treated due to concerns regarding additional toxicity. Furthermore, there is little evidence that routine pelvic radiotherapy in MIBC [8], [9] confers any benefit, despite evidence from surgical series showing significant rates of micro-metastases [10], [11].

This leaves a significant gap in treatment options for NPBC patients who are not suitable for surgery due to either comorbidities or personal choice. It could also be suggested that in patients with NPBC where the prognosis is poor, a less invasive approach with chemoradiation might be a more attractive alternative to surgery.

Advances in radiotherapy techniques have allowed more conformal approaches to be used, suggesting that nodal treatment could be delivered with acceptable toxicity. The Intensity-modulated Pelvic Node and Bladder Radiotherapy (IMPART) trial was designed to assess the feasibility of delivering intensity-modulated radiotherapy (IMRT) to the bladder and pelvic nodes, and to assess the clinical outcomes of patients treated.

## Materials and Methods

### Study Design

In this single-centre phase II study, patients with NPBC or high-risk node-negative bladder cancer (NNBC) received IMRT to the bladder and pelvic nodes. 13/38 (34.2%) patients were recruited retrospectively, having completed identical treatment to that offered within the trial before the study opened. To minimise potential bias, the retrospective group consisted of consecutively treated patients, with none excluded. The study and inclusion of the retrospective subset were approved by our institutional research committee and the local research ethics committee.

The primary end point was to assess the feasibility of meeting predetermined dose constraints. Secondary end points included assessment of acute toxicity, late toxicity at 1 year after treatment, pelvic relapse-free survival, distant relapse-free survival, overall survival and bladder cancer-specific survival.

Pelvic relapse-free survival was defined as the rate of survival free of recurrent disease (superficial and/or invasive) in the pelvic nodes or bladder.

### Eligibility

Eligible patients were aged 18 years or over with histopathologically proven MIBC and either (i) radiological or pathological evidence of pelvic nodal metastases or (ii) a high risk of nodal involvement, i.e. radiological or pathological T3b/T4 disease, or disease with high-risk pathology, e.g. small cell histology. Patients with pelvic relapse after radical cystectomy were also eligible.

Patients were required to have had a previous maximal transurethral resection of bladder tumour. Neoadjuvant chemotherapy was permitted.

Exclusion criteria included unsuitability for radical radiotherapy treatment, previous pelvic radiotherapy, inflammatory bowel disease or small bowel disease, bilateral hip replacements, other malignancy (except basal cell carcinoma, fully treated *in situ* cervical carcinoma or incidental prostate carcinoma) in the past 2 years that might affect treatment assessment.

All prospectively recruited patients provided written informed consent. For the retrospective cohort, written consent was obtained from those who were alive.

### Radiotherapy Planning

Patients underwent a radiotherapy planning scan in the supine position with an empty bladder, Combifix immobilisation and arms positioned across the chest. Slices (2.5 mm) were acquired from the cranial edge of the L3 vertebra to 1 cm below obturator foramina on a large-bore computed tomography scanner (Philips, Cleveland, OH, USA). Images were exported to a planning system (Pinnacle, Philips Medical, Madison, WI, USA) for target volume outlining.

### Radiotherapy Volumes

Four clinical target volumes (CTVs) were outlined: CTV__whole bladder_, CTV__pelvic lymph nodes_, CTV__tumour bed_ and CTV__involved lymph nodes_. Diagnostic imaging was used to assist outlining. Involved lymph nodes were determined by radiologist review of initial pre-treatment imaging and included all pathologically enlarged lymph nodes as per computed tomography size criteria [12]. Details of radiotherapy planning guidelines used are provided in the Supplementary Material.

**Fig 1:**
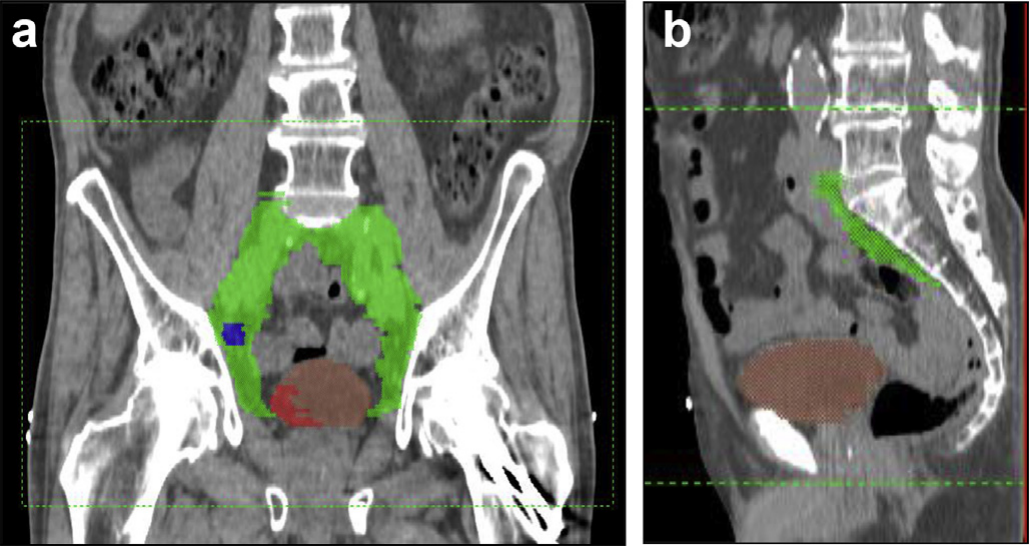
Examples of clinical target volumes (CTVs). (a) A coronal computed tomography slice illustrating CTV__whole bladder_ in brown, CTV__pelvic lymph nodes_ in green, CTV__tumour bed_ in red and CTV__involved lymph node_ in blue. (b) A sagittal computed tomography slice showing CTV__whole bladder_ and CTV__pelvic lymph nodes_.

Anisotropic margins were applied to CTV1 as per previous work [13]. Our institution standard of 0.5 cm was applied to the lymph node volumes. A 1 cm uniform margin was applied to the tumour bed (CTV3) to ensure coverage and to take into account possible tethering of the bladder wall at the site of disease. Table 1 summarises the volumes defined and expansion margins. Figure 1 illustrates the volumes created. The organs at risk included the rectum, other bowel and femoral heads. Table 2 summarises the predetermined dose constraints set.

**Table 1:** Clinical target volume (CTV) to planning target volume (PTV) margin expansions

	CTV1 → PTV1 (whole bladder)	CTV2 → PTV2 (pelvic lymph nodes)	CTV3 → PTV3 (bladder tumour bed)	CTV4 → PTV4 (involved pelvic lymph nodes)
Anterior	1.5 cm	0.5 cm	1.0 cm	0.5 cm
Posterior	1.0 cm	0.5 cm	1.0 cm	0.5 cm
Lateral	0.5 cm	0.5 cm	1.0 cm	0.5 cm
Superior	1.5 cm	0.5 cm	1.0 cm	0.5 cm
Inferior	0.5 cm	0.5 cm	1.0 cm	0.5 cm

**Table 2:** Predetermined dose constraints

Organ at risk	Dose	Maximum volume (% or cm^3^)
Rectum	30 Gy	80%
	50 Gy	60%
	60 Gy	50%
	65 Gy	30%
	70 Gy	15%
	75 Gy	5%
Femoral heads	50 Gy	50%
Other bowel	V45	139 cm^3^ optimal (209 cm^3^ mandatory)
	V50	122 cm^3^ optimal (183 cm^3^ mandatory)
	V55	105 cm^3^
	V60	84 cm^3^
	V65	26 cm^3^

### Radiotherapy Planning

All subjects were individually inverse-planned using five co-planar IMRT beams with gantry angles of 30, 95, 180, 265 and 330°. The PTV__whole bladder_ (excluding PTV__tumour bed_) and PTV__pelvic lymph nodes_ (excluding PTV__involved lymph nodes_) were prescribed to 52 Gy in 32 fractions, PTV__tumour bed_ was prescribed to 64 Gy in 32 fractions and PTV__involved lymph nodes_ was prescribed to 60 Gy in 32 fractions. The plan objectives were for the PTV D99 (i.e. the dose received by 99% of the PTV), D95 and D50 to be 90, 95 and 100% of the prescribed dose, respectively.

Where plans failed to meet PTV objectives and/or optimal organ at risk constraints, the target volumes and dose distributions were reviewed to produce a clinically acceptable option. Doses to the whole bladder and tumour bed were never compromised. Where optimal bowel constraints were still not met, 50% additional volume at the V45 and V50 levels were accepted, providing the V55, V60 and V65 constraints were not exceeded. If this was still not achievable, patients were treated to a nodal dose (PTV2) of 48 Gy in 32 fractions.

### Radiotherapy Delivery

Treatment was delivered daily with cone-beam computed tomography imaging acquired using a no action level offline protocol. Cone-beam computed tomography images were registered to the computed tomography scan using bony anatomy and a systematic correction carried out after the first three fractions. Weekly images were reviewed with a tolerance of 3 mm. Concomitant chemotherapy was recommended after publication of the BC2001 results [8]. Patients were reviewed weekly during treatment.

### Toxicity and Response Assessment

Patients were reviewed weekly during treatment and acute toxicities recorded using CTCAE v3.0. After radiotherapy, patients were reviewed at 4, 8 and 12 weeks. Late toxicity was graded using Radiation Therapy Oncology Group criteria from week 12. Patients underwent a rigid cystoscopy at week 12. Patients were then clinically reviewed every 6 months up to 3 years after radiotherapy, then annually to 5 years. A chest X-ray was carried out at 6, 18, 30, 36, 48 and 60 months. Computed tomography of the chest/abdomen/pelvis was carried out at 12 and 24 months. Flexible cystoscopy was carried out at 6, 12, 18, 24 months, and then annually to 5 years.

### Statistics

The sample size was based on the hypothesis that the true rate of meeting dose constraints would be in the region of 70%, and for the feasibility end point we intended to show that >50% of patients would meet all dose constraints. Using a Simon two-stage design with a one-sided test at significance level 0.05, initial recruitment was set at 23 patients, with a further 14 to be recruited if at least 13/23 met the constraints. The end point would be considered to have been met if at least 24/37 met the dose constraints.

Toxicity data were analysed in terms of prevalence and overall frequencies. Survival and recurrence-free estimates were calculated using Kaplan–Meier analysis on an intention to treat basis. The follow-up time was calculated from the start date of radiotherapy to the date of death (event) or the date last known to be alive (censored).

An exploratory analysis was carried out to compare acute toxicity profiles in patients receiving radiotherapy alone versus chemoradiation, with significance assessed using a Fisher's exact test. A further exploratory analysis using Kaplan–Meier methods was carried out to look at pelvic relapse-free and overall survival in patients with NPBC versus NNBC. The Cox proportional hazard model was used to calculate hazard ratios with a 95% confidence interval. Data were analysed using STATA 13.1 for Windows.

## Results

Between June 2009 and November 2012, 38 patients were recruited. Patient characteristics are shown in Table 3. Of the 16/38 (42.1%) patients with NNBC, 11/16 (68.8%) had T3/4 disease, 4/16 (25.0%) had neuroendocrine differentiation and 1/16 (6.3%) had bulky multifocal disease.

**Table 3:** Patient characteristics

Gender *n* (%)	Female	7	(18.4)
	Male	31	(81.6)
Age (years)	Median	70.7	
	Range	47–88	
Clinical T stage *n* (%)	T2	11	(28.9)
	T3	18	(47.4)
	T4	6	(15.8)
	Prior cystectomy	3	(7.9)
Pathological T stage *n* (%)	pT1	2	(5.3)
	pT2	30	(78.9)
	pT3a/b	0	(0)
	pT4	2	(5.3)
	Prior cystectomy	3	(7.9)
	Not known	1	(2.6)
Clinical N stage *n* (%)	N0	16	(42.1)
	N1	9	(23.7)
	N2	9	(23.7)
	N3	4	(10.5)
Histology∗*n* (%)	Transitional cell	33	(86.8)
	Small cell	3	(7.9)
	Squamous cell	1	(2.6)
	Not known	1	(2.6)
Neoadjuvant chemotherapy *n* (%)	Yes	31	(81.6)
	No	7	(18.4)
Concurrent chemotherapy *n* (%)	Yes	18	(47.4)
	No	20	(52.6)

∗ All were high grade.

### Primary End Point

The radiotherapy plans of 29/38 (76.3%) patients met all predetermined dose constraints. The remaining patients failed to meet the ‘other bowel’ dose constraint. A clinically acceptable option was achievable in 5/9 (55.6%) patients without dose reduction; 3/9 (33.3%) patients required the prescribed dose to PTV2 to be reduced to 48 Gy in 32 fractions. In one case, the dose constraints were not achievable even at a reduced dose prescription.

Overall, 97% (37/38) of patients had radiotherapy planned as per the study protocol. A total of 35/38 (92.1%) patients completed radiotherapy as planned.

### Secondary End Points

#### Acute Toxicity

One patient died before starting radiotherapy and so was excluded from the toxicity analysis. As three patients had had a previous cystectomy, urinary toxicity data were collected from 34 patients. The results are summarised in Table 4.

**Table 4:** Acute toxicity

	Acute toxicity (CTCAE v3)	Grade 0	Grade 1	Grade 2	Grade 3	Grade 4	Data unavailable∗
Gastrointestinal	Diarrhoea	10 (27.0%)	17 (46.0%)	8 (21.6%)	1 (2.7%)	0 (0.0%)	1 (2.7%)
	Proctitis	25 (67.6%)	5 (13.5%)	6 (16.2%)	0 (0.0%)	0 (0.0%)	1 (2.7%)
	Abdominal pain	18 (48.6%)	17 (45.9%)	1 (2.7%)	0 (0.0%)	0 (0.0%)	1 (2.7%)
	Nausea	25 (68.0%)	10 (27.0%)	1 (2.7%)	0 (0.0%)	0 (0.0%)	1 (2.7%)
	Vomiting	33 (89.2%)	3 (8.1%)	0 (0.0%)	0 (0.0%)	0 (0.0%)	1 (2.7%)
	Anorexia	21 (56.8%)	7 (18.9%)	7 (18.9%)	1 (2.7%)	0 (0.0%)	1 (2.7%)
	Gastrointestinal overall	4 (11.0%)	14 (28.6%)	16 (43.2%)	2 (5.4%)	0 (0.0%)	1 (2.7%)
Genitourinary	Urinary frequency/urgency	3 (8.8%)	13 (38.2%)	11 (32.4%)	6 (17.6%)	0 (0.0%)	1 (2.9%)
	Cystitis	7 (20.6%)	12 (32.4%)	14 (41.2%)	0 (0.0%)	0 (0.0%)	1 (2.9%)
	Urinary incontinence	22 (64.7%)	7 (20.6%)	3 (8.8%)	1 (2.9%)	0 (0.0%)	1 (2.9%)
	Retention/hesitancy	20 (58.8%)	11 (32.4%)	1 (2.9%)	1 (2.9%)	0 (0.0%)	1 (2.9%)
	Bladder spasm	26 (76.5%)	6 (17.6%)	1 (2.9%)	0 (0.0%)	0 (0.0%)	1 (2.9%)
	Genitourinary overall	2 (5.8%)	8 (23.5%)	16 (47.1%)	7 (20.6%)	0 (0.0%)	1 (2.9%)
Other	Fatigue	6 (16.2%)	18 (48.6%)	11 (29.7%)	0 (0.0%)	1 (2.7%)	1 (2.7%)
	Haemoglobin	4 (10.8%)	23 (62.2%)	9 (24.3%)	0 (0.0%)	0 (0.0%)	1 (2.7%)

∗ One patient had a 6-week treatment break due to a fractured hip and resumed a hypofractionated schedule off-study. Toxicity data were therefore not collected.

Diarrhoea and urinary frequency/urgency were the most commonly reported adverse events, affecting 26/37 (70.3%) and 28/34 (82.4%) patients, respectively. Grade 1/2 gastrointestinal and genitourinary adverse events were reported by 30/37 (81.1%) and 24/34 (70.6%) patients, respectively. The grade 3 gastrointestinal toxicity rate was 5.4% and the grade 3 genitourinary toxicity rate was 20.6%. There were no grade 4 gastrointestinal or genitourinary adverse events. An exploratory comparison of the maximum toxicity reported by patients receiving radiotherapy alone or chemoradiation is shown in the Supplementary Material.

#### Late Toxicity

Late toxicity data were collected at a minimum of one time point from 30/38 (78.9%) patients.

Data were unavailable for 8/38 (21.1%) patients. Of those with missing data, 4/8 (50%) died before 12 weeks, 2/8 (25%) relapsed with metastatic disease before 12 weeks and subsequently died, 1/8 (12.5%) withdrew from the study and 1/8 (12.5%) was treated off-protocol.

At 1 year, the grade 3 late toxicity rate was 5%, with one patient reporting grade 3 cystitis and haematuria. At 2 years, there was no reported grade 3 or 4 toxicity.

### Overview of Relapse Patterns

At a median follow-up of 5.2 years, 27/38 (71.1%) patients had disease recurrence. At first relapse, 15/38 (39.5%) patients had locoregional disease only, 5/38 (13.2%) had both locoregional and distant recurrence and 7/38 (18.4%) presented with distant metastases only; 9/38 (23.7%) patients showed no evidence of disease recurrence and 2/38 (5.2%) patients died before/during radiotherapy from unrelated causes.

**Fig 2:**
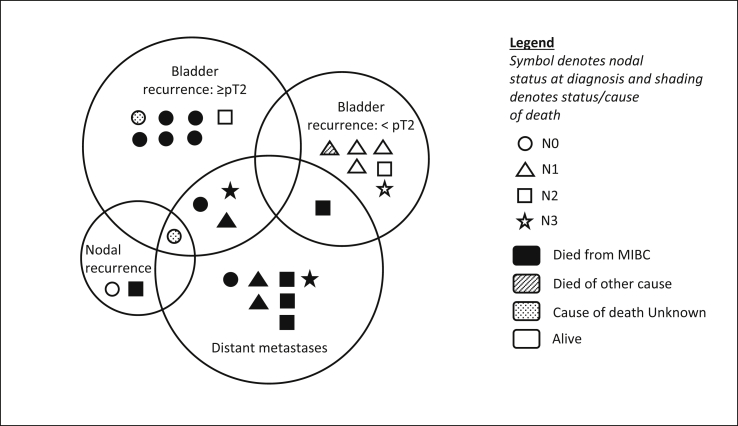
Proportional Venn diagram illustrating disease patterns at first relapse and survival status at a median follow-up of 5.2 years.

**Fig 3:**
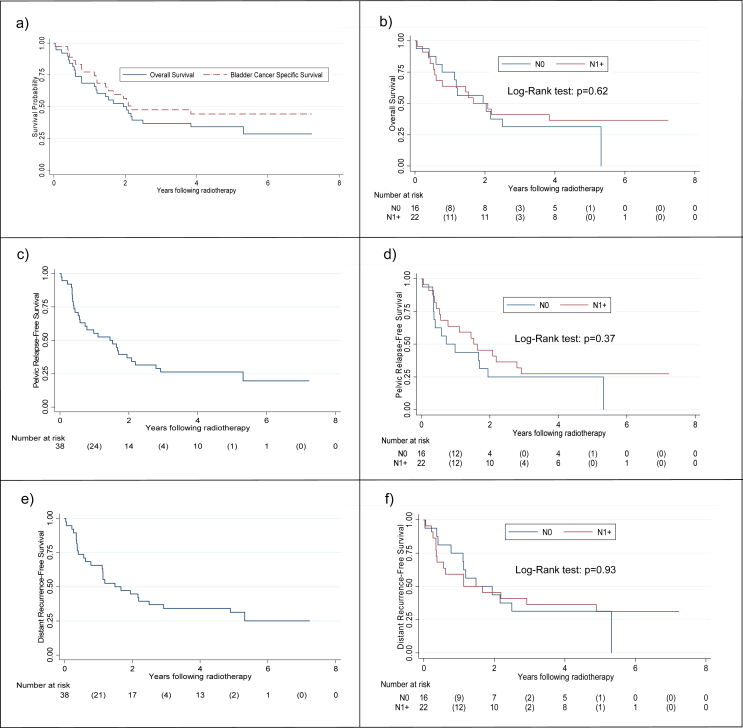
Kaplan–Meier curves showing: (a) overall survival and bladder cancer-specific survival, (b) overall survival according to nodal status, (c) pelvic relapse-free survival, (d) pelvic relapse-free survival according to nodal status, (e) distant recurrence-free survival and (f) distant recurrence-free survival according to nodal status.

Four of 15 (26.7%) patients with initial locoregional only relapse subsequently went on to develop distant metastases. Disease patterns at first relapse are summarised in Figure 2. Figure 3 shows the Kaplan–Meier survival curves.

#### Pelvic Relapse

Twenty of 38 (52.6%) patients had locoregional disease at first relapse, of whom a quarter had concurrent distant metastases.

Of the 35 patients with an intact bladder at treatment, 11/35 (31.4%) developed muscle-invasive bladder recurrence (4/11 with concurrent metastases, including one with additional nodal relapse) and 7/35 (20.0%) developed superficial disease; two of whom subsequently developed muscle-invasive disease 11 and 12 months later. Two of 38 (5.2%) patients developed isolated pelvic nodal relapse. Ten of 11 (90.9%) invasive bladder relapses occurred at the same site as the original disease.

One-, 2- and 5-year locoregional relapse-free survival rates were 55, 37 and 26%, respectively.

An exploratory analysis comparing locoregional control in NPBC and NNBC patients did not show a significant difference (*P* = 0.37).

Salvage cystectomy was carried out in five patients at a median of 259 days after radiotherapy (range 146–689). Three of five (80%) patients had ≥ pT3 disease, 1/5 (20%) had pT1b and Carcinoma *in situ* (CIS) disease and 1/5 (20%) had a complete pathological response after re-challenge with carboplatin and etoposide. The bladder preservation rate for the cohort overall was 85.7% (30/35).

#### Distant Relapse

Twelve of 38 (31.6%) patients first relapsed with distant metastatic disease, five of whom (41.7%) had concurrent locoregional recurrence.

Two patients initially relapsing with CIS only subsequently went on to develop distant metastatic disease at 1.4 and 4 years after recurrence. A further two patients with muscle-invasive bladder only disease at first relapse went on to develop distant metastatic disease 10.5 and 9 months later. Therefore, a total of 16/38 (42.1%) patients developed distant metastases after radiotherapy, 11 of whom had NPBC; representing 50% of the 22 NPBC patients.

One-, 2- and 5-year distant metastasis-free survival rates were 66, 45% and 31%, respectively.

An exploratory analysis comparing distant metastasis-free survival between NNBC and NPBC patients showed no significant difference (*P* = 0.93).

### Overall Survival

The median overall survival was 1.9 years (95% confidence interval 1.1–3.8) with 1-, 2- and 5-year overall survival rates of 68, 50 and 34%, respectively.

An exploratory analysis comparing overall survival in NNBC and NPBC patients showed no significant difference (*P* = 0.62).

### Bladder Cancer-specific Survival

The median bladder cancer-specific survival was 2.2 years (95% confidence interval 1.5 – not reached) with 1-, 2- and 5-year bladder cancer-specific survival rates of 78, 57 and 44%, respectively.

## Discussion

IMPART has shown that delivering IMRT to the pelvic nodes and bladder is feasible in terms of meeting predetermined dose constraints. This supports the recent work of Sondergaard *et al.* [14], who reported statistically significant normal tissue sparing in a cohort of 16 NNBC patients when IMRT was used to treat the bladder and the pelvic lymph nodes compared with a conformal sequential boost technique. Furthermore, a subset of our cohort achieved long-term disease control and overall toxicity levels seem to be in line with those reported in other studies where the bladder and the pelvic lymph nodes were treated [9], [14].

The acute and late gastrointestinal toxicity rates are of particular interest given the large radiation fields required to encompass the pelvic nodes and the proximity of bowel to the bladder and pelvic nodal volumes. Although 70% of our patients reported diarrhoea at some point, most cases were limited to grade 1/2 in severity and only one patient reported grade 3 diarrhoea. Our grade 1/2 gastrointestinal toxicity rate of 81.1% compares favourably with an IMRT study [14] where equivalent rates of 75% were reported in a cohort of NNBC patients receiving 48 Gy in 30 fractions to the pelvic nodes without concomitant chemotherapy. Our slightly higher toxicity rates are probably due to the difference in nodal dose delivered and the fact that 50% of our cohort also received concomitant chemotherapy, which is associated with increased bowel toxicity [8].

It is interesting to note that although concerns regarding the inclusion of the pelvic nodes in the radiation field have been primarily based on concerns regarding bowel toxicity, we found that the grade 3/4 urinary toxicity rates were higher than those reported for gastrointestinal adverse effects. We suggest, therefore, that our results are encouraging with regards to bowel toxicity and support previous work suggesting that existing bowel constraints are too conservative in the setting of bladder cancer [15].

A review of the relapse patterns seen in this cohort is important in defining how to improve treatment. The rate of pelvic nodal relapse was low and actually little different to the rate seen after bladder-only radiotherapy (<6%) [8], suggesting that the nodal doses used in this study are sufficient to attain control in most patients. Local relapse in these high-risk patients, both invasive and non-invasive, however, was more common. Only half of patients received concurrent chemotherapy, which would now be considered as best practice as it was shown to significantly reduce locoregional recurrence in the BC2001 trial. Of those not receiving concurrent chemotherapy, 12 patients were treated before publication of BC2001 changed our routine practice and six patients had comorbidities precluding concurrent treatment. These data, however, do lend support to investigating strategies to improve local control. This might include additional concomitant therapies or dose-escalation strategies to the tumour bed. We have recently reported results of dose escalating the tumour dose in standard-risk patients to 70 Gy/32 fractions [16] and are currently studying this approach in NPBC patients.

The high incidence of distant metastatic relapse, despite most patients receiving neoadjuvant chemotherapy, after radical treatment demonstrated in this cohort emphasises the poor prognosis and systemic nature of the disease and highlights the ongoing need for more efficacious systemic treatment options. Exploration of additional treatment approaches, such as immune checkpoint (PD-1, PDL-1) inhibitors, might be of interest.

Despite this, recurrence-free survival and overall survival seem to plateau after 3 years, with a subset of patients showing long-term disease control. Our 5-year overall survival rate of 34% is comparable with the 5-year cancer-specific survival of 29.2% in 149 NPBC patients undergoing neoadjuvant chemotherapy and surgery [2].

Our exploratory analysis showed no significant difference in outcome between NPBC and NNBC. However, over two-thirds of those with NNBC had T3/4 disease and 19% had small cell histology. Both of these features are associated with a poor prognosis and so the lack of difference between the subgroups is perhaps not so surprising.

The limitations of this study include the fact that this was a small and heterogeneous cohort, which included NPBC, NNBC, post-cystectomy and retrospectively recruited patients, thus limiting the power of any clinical outcome analysis. However, the primary aim of this study was to assess the feasibility of delivering IMRT to the bladder and pelvic nodes, and this was met.

## Conclusion

IMRT to the bladder and pelvic nodes in MIBC is feasible in terms of meeting dose constraints and shows an acceptable adverse effect profile. Although many centres have traditionally adopted a palliative approach to NPBC, our results support existing surgical data demonstrating that a subset of NPBC patients can achieve long-term control after radical treatment and IMPART suggests that chemoradiation could offer an alternative to surgery for such patients. However, further work is required to fully evaluate the role of nodal irradiation in MIBC. Identifying ‘treatment responders’ will be key and will probably rely on both clinicopathological features and translational biomarkers. There remains, however, a high risk of developing distant disease in MIBC and so optimisation of local treatment alone is insufficient. Strategies to target distant metastases and, if possible, prevent the development of metastases remain crucial in the management of this poor-prognosis disease.

## Conflicts of Interest

D. Dearnaley reports grants from Cancer Research UK and the National Institute for Health Research during the conduct of the study and personal fees from Takeda, Amgen, Astellas, Sandoz and Janssen Pharma outside the submitted work. In addition, D. Dearnaley has patent EP1933709Bi issued and is on the Institute of Cancer Research's Rewards to Inventors list for abiraterone acetate. R.A. Huddart is a member of the Elekta MR-Linac Consortium and reports personal fees from Janssen, MSD, Roche and Bristol-Myers Squibb outside the submitted work. S. Hafeez reports non-financial support from Elekta and Merck Sharp & Dohme.

## References

[1] Gschwend J.E., Dahm P., Fair W.R. (2002). Disease specific survival as endpoint of outcome for bladder cancer patients following radical cystectomy. Eur Urol.

[2] Stein J.P., Lieskovsky G., Cote R., Groshen S., Feng A.C., Boyd S. (2001). Radical cystectomy in the treatment of invasive bladder cancer: long-term results in 1,054 patients. J Clin Oncol.

[3] Lerner S.P., Skinner D.G., Lieskovsky G., Boyd S.D., Groshen S.L., Ziogas A. (1993). The rationale for en bloc pelvic lymph node dissection for bladder cancer patients with nodal metastases: long-term results. J Urol.

[4] Shariat S.F., Karakiewicz P.I., Palapattu G.S., Lotan Y., Rogers C.G., Amiel G.E. (2006). Outcomes of radical cystectomy for transitional cell carcinoma of the bladder: a contemporary series from the Bladder Cancer Research Consortium. J Urol.

[5] Mills R.D., Turner W.H., Fleischmann A., Markwalder R., Thalmann G.N., Studer U.E. (2001). Pelvic lymph node metastases from bladder cancer: outcome in 83 patients after radical cystectomy and pelvic lymphadenopathy. J Urol.

[6] Madersbacher S., Hochreiter W., Burkhard F., Thalmann G.N., Danuser H., Markwalder R. (2003). Radical cystectomy for bladder cancer today – a homogeneous series without neoadjuvant therapy. J Clin Oncol.

[7] Meijer R.P., Mertens L.S., van Rhijn B.W., Bex A., van der Poel H.G., Meinhardt W. (2014). Induction chemotherapy followed by surgery in node positive bladder cancer. Urology.

[8] James N.D., Hussain S.A., Hall E., Jenkins P., Tremlett J., Rawlings C. (2012). Radiotherapy with or without chemotherapy in muscle-invasive bladder cancer. N Engl J Med.

[9] Tunio M.A., Hashmi A., Qayyum A., Mohsin R., Zaeem A. (2012). Whole-pelvis or bladder-only chemoradiation for lymph node-negative invasive bladder cancer: single-institution experience. Int J Radiat Oncol Biol Phys.

[10] Gray P.J., Lin C.C., Jemal A., Shipley W.U., Fedewa S.A., Kibel A.S. (2014). Clinical-pathologic stage discrepancy in bladder cancer patients treated with radical cystectomy: results from the national cancer data base. Int J Radiat Oncol Biol Phys.

[11] Goldsmith B., Baumann B.C., He J., Tucker K., Bekelman J., Deville C. (2014). Occult pelvic lymph node involvement in bladder cancer: implications for definitive radiation. Int J Radiat Oncol Biol Phys.

[12] Vinnicombe S.J., Norman A.R., Nicolson V., Husband J.E. (1995). Normal pelvic lymph nodes: evaluation with CT after bipedal lymphangiography. Radiology.

[13] Lalondrelle S., Huddart R., Warren-Oseni K., Hansen V.N., McNair H., Thomas K. (2011). Adaptive-predictive organ localization using cone-beam computed tomography for improved accuracy in external beam radiotherapy for bladder cancer. Int J Radiat Oncol Biol Phys.

[14] Sondergaard J., Hoyer M., Petersen J.B., Wright P., Grau C., Muren L.P. (2009). The normal tissue sparing obtained with simultaneous treatment of pelvic lymph nodes and bladder using intensity-modulated radiotherapy. Acta Oncol.

[15] McDonald F., Waters R., Gulliford S., Hall E., James N., Huddart R.A. (2015). Defining bowel dose volume constraints for bladder radiotherapy treatment planning. Clin Oncol.

[16] Hafeez S., Warren-Oseni K., McNair H.A., Hansen V.N., Jones K., Tan M. (2016). Prospective study delivering simultaneous integrated high-dose tumor boost (</=70 Gy) with image guided adaptive radiation therapy for radical treatment of localized muscle-invasive bladder cancer. Int J Radiat Oncol Biol Phys.

