# Supplementary material for: The Intensity-Modulated Pelvic Node and Bladder Radiotherapy (IMPART) Trial: A Phase II Single-Centre Prospective Study
Source: Clin Oncol (R Coll Radiol). 2020 Feb;32(2):93–100. doi: 10.1016/j.clon.2019.07.017 (PMC6966321; doi:10.1016/j.clon.2019.07.017)
Supplement: Multimedia component 1 [file mmc1.docx]

Supplementary Material

1. Radiotherapy planning guidelines

**Volume definitions**

CTVs

- *CTV1 = Whole bladder*

Delineated by the outer bladder wall and includes extravesical disease. The inferior limit of the bladder includes 1.5cm of prostatic urethra in all male patients, and 1cm length of urethra in females where there is a bladder base tumour or diffuse CIS.

This volume will not be included in post-cystectomy patients.

- *CTV2 = Pelvic lymph nodes*

This includes the following nodal areas: the pre-sacral chain to bottom of S3, the pre-sciatic nodes, the external iliac nodes and the internal iliac nodes, including the hypogastric-obturator complex).

- *CTV3 = Bladder tumour bed*

The bladder tumour bed is the site of original disease as seen on pre-chemotherapy imaging.

- *CTV4 = Involved pelvic lymph nodes*

Involved pelvic lymph nodes are determined at radiologist review of pre-chemotherapy imaging, and include all pathologically enlarged lymph nodes determined by CT size criteria (≥10mm in short axis diameter).

Organs at Risk

- *Rectum*

The circumference of the rectum should be outlined in its entirety, to include the faecal contents. Outlining should extend from the anus (usually at level of the ischial tuberosities or 1cm below the lower margin of the PTV – whichever is more inferior) to the rectosigmoid junction. The rectosigmoid junction can usually be identified on the CT slice where the bowel turns anteriorly and to the left. The overall length of the rectum is typically 10-12cm.

- *Bowel*

The entire bowel visible on relevant levels of the planning scan will be outlined and included in the analysis. The outlining will include the small bowel, the large bowel and the sigmoid colon, down to the level of the rectosigmoid junction. The superior extent of outlining should be 2cm beyond the superior extent of the PTV_2_.

- *Right and left femoral heads*

The femoral heads are outlined to the bottom of the curvature of their heads.

# Outlining protocol for CTV_2_ (pelvic lymph nodes)

Lymph nodes are not readily identifiable on the planning CT scans. The relationship between the nodes and the vasculature is therefore used to ensure that the nodes are included within the CTV_2_ [31]. Before outlining it is advisable to identify the various structures – especially the vessels and bowel. This can be done with more confidence by following their course over several scans.

1. Sacral promontory to bottom of anterior extent of S3/4 junction – pre-sacral and upper pelvic nodes.

- At the sacral promontory, the outline starts at the anterior extent of iliac vessels. It follows the anterior wall of the vessels and laterally follows the plane between vessels and psoas muscle (or the medial border of the psoas muscle if there is a distinct fat plane between the vessels and psoas). Posteriorly the outline extends onto the sacrum and stops at the anterior extent of the sacrum.
- To cross the midline anteriorly, three situations occur. Firstly, if there is a vessel crossing the midline, the outline should follow the anterior extent of this vessel, and continue anterior to the wall of the vessel until it joins the starting point. Secondly, if there is no vessel crossing the midline, the anterior extent of the outline crossing the midline should be 15mm anterior to the sacrum. This will include the pre-sacral nodes. Thirdly, if there is bowel in this pre-sacral space it should be specifically excluded from the volume. In this case the outline follows the posterior wall of the bowel. The first outline should now be complete.
- The outline on subsequent scans follows the same path until it reaches the anterior aspect of the S3/4 junction apart from one point. As the anterior extent of the sacrum becomes almost horizontal, the lateral extent of the sacrum is defined by the sacro-iliac joints and usually corresponds to the point where the psoas muscle meets the pelvic bone. A corner is created which marks the postero-lateral extent of the outline. The lateral outline, running between the iliac vessels and the psoas should follow the medial border of psoas posteriorly onto the bone, and the outline should then continue medially.
- Proceeding caudally, the sacrum has hollows, which correspond to the exit foramina of the sacral nerves, and should be included within the volume (i.e. the outline continues to follow the anterior extent of the bone). The pyriformis muscle lies anterior to the sacrum and becomes bulkier caudally, its anterior border becomes the posterior border of the outline (i.e. it is excluded from the volume).
- The bottom of this section corresponds to the anterior extent of the S3/4 junction, which is identified from the sagittal scout film and approximates to the bottom of the sacro-iliac joints.

1. Mid pelvic nodes.

- At the anterior extent of S3/4 junction (defined from lateral scout view) the outline now excludes the remaining inferior extent of the pre-sacral space.
- The outline starts at the anterior extent of the external iliac vessels. Laterally it follows initially the medial edge of the psoas muscle, and then the medial wall of the pelvis. The posterior edge of the ilium marks the anterior portion of the sciatic notch. The pre-sciatic nodes (also known as the internal pudendal nodes), accompanying vessels (continuation of the internal iliac vessels) and the sciatic nerve lie in this area. The outline extends down from the bony pelvis and passes lateral to these structures (i.e. they are included). The outline runs along the visible musculature (obturator externus), which forms the postero-lateral border of the volume. The posterior extent of the outline runs along the most posterior of the previously mentioned structures. This usually involves outlining as much as 2/3^rds^ of the sciatic foramen. The outline then runs up the medial border of these structures and should then become the medial border of the internal iliac vessels.
- The next part of the medial outline is variable. The follows the medial border of the external iliac vessels. Between the internal and external iliac vessels, the outline is drawn to include branches of the internal iliac vessels but excludes bowel.
- The anterior extent of the outline continues to be the anterior wall of the external iliac vessels, but it should not extend more than about 2cm anterior to the most anterior point of the bony pelvis and should include both artery and vein. Eventually both vessels are anterior and lateral to the pelvic brim, as they descend into the groin. At this point, the anterior extent of the outline corresponds to the antero-medial point of the bony pelvis and usually approximates to the level of the acetabulum.
- Caudally, the medial edge of the outline follows the medial edge of the vessels. The outlines stay separate.

1. Lower pelvic nodes

- The anterior extent of the outline continues to be the antero-medial bony pelvis. The lateral outline follows the medial edge of the obturator internus muscle and posteriorly the bony pelvis. When the structures in the pre-sciatic notch become invisible, the posterior extent of the outline becomes the posterior point of the ilium. The medial outline follows the medial border of the small vessels.
- As the superior pubic ramus starts to appear, the nodal volume reduces further in size. The anterior extent of the nodal volume becomes the anterior extent of the vessels on the pelvic sidewalls, and the posterior extent becomes the posterior extent of the vessels. The lateral border remains the musculature and bony pelvis. The outline stops extending to the pelvic side walls 0.5-1 cm above the top of the acetabulum.

1. Exploratory Analysis of Toxicity Profiles Between Patients Receiving Radiotherapy Alone or Chemoradiation

|  | Acute Toxicity  (CTCAE v3) | Grade | Radiotherapy Alone  (n = 19^%^) | Chemoradiation  (n = 18) | p-value (Fisher’s Exact)^$^ |
| --- | --- | --- | --- | --- | --- |
| GI | Diarrhoea | G0 | 8 | 2 | P=0.02 |
|  |  | G1 | 9 | 8 |  |
|  |  | G2 | 1 | 7 |  |
|  |  | G3 | 0 | 1 |  |
|  |  | G4 | 0 | 0 |  |
|  |  | ND | 1 | 0 |  |
|  | Proctitis | G0 | 14 | 11 | P=0.60 |
|  |  | G1 | 2 | 3 |  |
|  |  | G2 | 2 | 4 |  |
|  |  | G3 | 0 | 0 |  |
|  |  | G4 | 0 | 0 |  |
|  |  | ND | 1 | 0 |  |
|  | Abdominal pain | G0 | 10 | 8 | P = 0.51 |
|  |  | G1 | 7 | 10 |  |
|  |  | G2 | 1 | 0 |  |
|  |  | G3 | 0 | 0 |  |
|  |  | G4 | 0 | 0 |  |
|  |  | ND | 1 | 0 |  |
|  | Nausea | G0 | 15 | 10 | P=0.06 |
|  |  | G1 | 2 | 8 |  |
|  |  | G2 | 1 | 0 |  |
|  |  | G3 | 0 | 0 |  |
|  |  | G4 | 0 | 0 |  |
|  |  | ND | 1 | 0 |  |
|  | Vomiting | G0 | 17 | 16 | P = 1.00 |
|  |  | G1 | 1 | 2 |  |
|  |  | G2 | 0 | 0 |  |
|  |  | G3 | 0 | 0 |  |
|  |  | G4 | 0 | 0 |  |
|  |  | ND | 1 | 0 |  |
|  | Anorexia | G0 | 12 | 9 | P=0.52 |
|  |  | G1 | 2 | 5 |  |
|  |  | G2 | 3 | 4 |  |
|  |  | G3 | 1 | 0 |  |
|  |  | G4 | 0 | 0 |  |
|  |  | ND | 1 | 0 |  |
|  | GI OVERALL | G0 | 3 | 1 | P=0.25 |
|  |  | G1 | 9 | 5 |  |
|  |  | G2 | 5 | 11 |  |
|  |  | G3 | 1 | 1 |  |
|  |  | G4 | 0 | 0 |  |
|  |  | ND | 1 | 0 |  |
| GU^ | Urinary frequency/urgency | G0 | 3 | 0 | P=0.12 |
|  |  | G1 | 6 | 7 |  |
|  |  | G2 | 3 | 8 |  |
|  |  | G3 | 4 | 2 |  |
|  |  | G4 | 0 | 0 |  |
|  |  | ND | 1 | 0 |  |
|  |  | PC | 2 | 1 |  |
|  | Cystitis | G0 | 4 | 3 | P=0.90 |
|  |  | G1 | 6 | 6 |  |
|  |  | G2 | 6 | 8 |  |
|  |  | G3 | 0 | 0 |  |
|  |  | G4 | 0 | 0 |  |
|  |  | ND | 1 | 0 |  |
|  |  | PC | 2 | 1 |  |
|  | Urinary incontinence | G0 | 12 | 10 | P=0.29 |
|  |  | G1 | 4 | 3 |  |
|  |  | G2 | 0 | 3 |  |
|  |  | G3 | 0 | 1 |  |
|  |  | G4 | 0 | 0 |  |
|  |  | ND | 1 | 0 |  |
|  |  | PC | 2 | 1 |  |
|  | Retention/hesitancy | G0 | 12 | 8 | P= 0.03 |
|  |  | G1 | 2 | 9 |  |
|  |  | G2 | 1 | 0 |  |
|  |  | G3 | 1 | 0 |  |
|  |  | G4 | 0 | 0 |  |
|  |  | ND | 1 | 0 |  |
|  |  | PC | 2 | 1 |  |
|  | Bladder spasm | G0 | 16 | 10 | P=0.01 |
|  |  | G1 | 0 | 6 |  |
|  |  | G2 | 0 | 1 |  |
|  |  | G3 | 0 | 0 |  |
|  |  | G4 | 0 | 0 |  |
|  |  | ND | 1 | 0 |  |
|  |  | PC | 2 | 1 |  |
|  | GU OVERALL | G0 | 2 | 0 | P=0.46 |
|  |  | G1 | 4 | 4 |  |
|  |  | G2 | 6 | 10 |  |
|  |  | G3 | 4 | 3 |  |
|  |  | G4 | 0 | 0 |  |
|  |  | ND | 1 | 0 |  |
|  |  | PC | 2 | 1 |  |
| Other | Fatigue | G0 | 4 | 2 | P=0.74 |
|  |  | G1 | 8 | 10 |  |
|  |  | G2 | 5 | 6 |  |
|  |  | G3 | 0 | 0 |  |
|  |  | G4 | 1 | 0 |  |
|  |  | ND | 1 | 0 |  |
|  | Haemoglobin | G0 | 3 | 1 | P=0.37 |
|  |  | G1 | 12 | 11 |  |
|  |  | G2 | 3 | 6 |  |
|  |  | G3 | 0 | 0 |  |
|  |  | G4 | 0 | 0 |  |
|  |  | ND | 1 | 0 |  |
| ^%^ Patient treated off-study with hypofractionated schedule after 6-week treatment break due to hip fracture- toxicity data not collected (ND = not documented); ^GU toxicity collected from 34 patients with intact bladder (PC = prior cystectomy)  $ Using Bonferroni correction to adjust for multiple testing, a p-value of <0.00217 is considered statistically significant | | | | | |
